# RiboTagger: fast and unbiased 16S/18S profiling using whole community shotgun metagenomic or metatranscriptome surveys

**DOI:** 10.1186/s12859-016-1378-x

**Published:** 2016-12-22

**Authors:** Chao Xie, Chin Lui Wesley Goi, Daniel H. Huson, Peter F. R. Little, Rohan B. H. Williams

**Affiliations:** 1 Singapore Centre for Environmental Life Sciences Engineering, National University of Singapore, Singapore, 117456 Singapore; 2 Centre for Bioinformatics, Tuebingen University, Tuebingen, 72076 Germany; 3 Life Sciences Institute, National University of Singapore, Singapore, 117456 Singapore; 4 Department of Biochemistry, Yong Loo Lin School of Medicine, National University of Singapore, Singapore, 117596 Singapore; 5 Current address: Human Longevity Inc, Singapore, Singapore

**Keywords:** Microbial ecology, Short subunit ribosomal RNA, Microbial community profiling, Sequence analysis

## Abstract

**Background:**

Taxonomic profiling of microbial communities is often performed using small subunit ribosomal RNA (SSU) amplicon sequencing (16S or 18S), while environmental shotgun sequencing is often focused on functional analysis. Large shotgun datasets contain a significant number of SSU sequences and these can be exploited to perform an unbiased SSU--based taxonomic analysis.

**Results:**

Here we present a new program called RiboTagger that identifies and extracts taxonomically informative ribotags located in a specified variable region of the SSU gene in a high-throughput fashion.

**Conclusions:**

RiboTagger permits fast recovery of SSU-RNA sequences from shotgun nucleic acid surveys of complex microbial communities. The program targets all three domains of life, exhibits high sensitivity and specificity and is substantially faster than comparable programs.

## Background

Studying the composition and dynamics of microbial communities is a key problem in microbiome research and microbial ecology [1]. Traditionally, these studies have been based on isolating and sequencing short subunits of the 16S and 18S genes, present in bacteria or archea, and eukaryotes, respectively. Typically most studies now make use of amplicon sequencing to obtain such data from complex microbial communities [2, 3]. The PCR amplicon technique was very useful when sequencing power was limited, however, with the increasing power and complexity of the new generation of sequencing technologies, the broad advantages of amplicon sequencing are starting to be balanced by major limitations, which include PCR primer selection and amplification bias [4, 5]. In particular, no PCR primers are able to amplify all known bacterial taxonomic groups efficiently and uniformly [5], which leads to biased rRNA profiling analysis, and the use of short read technologies, notably Illumina, results in a complex, interdependent chain of technical decisions, that can heavily influence the subsequent community profiling results [6].

Within microbiome research, there is increasing use of whole community gDNA surveys (i.e. shotgun metagenomics), which offer, at theoretically, a less biased view of community composition than using from amplicon based methods, by eliminating dependency on 16S primers [7]. In practice however, the interpretation of shotgun metagenome data is heavily dependent on having access to reference genomes of community members, without which substantial limitations of interpretation may arise [8]. While the intended use of these shotgun data is typically to capture functional capacity of a community [9], or to permit member genome recovery [8], it has been recognized that whole commnuity shotgun surveys will of course contain a substantial number of reads derived from SSU-rRNA genes, and these can be exploited for the purposes of community profiling [10–17]. Similarly, when using total RNA metatranscriptome sequencing, rRNA often account for 95% of reads sequenced, and thus provides coverage of SSU diversity to great depth. Within this general area, a number of examples of this approach have been undertaken and several software implementations of this approach are now available [13, 16, 17], mostly based on the use of Hidden Markov Models to capture reads of SSU-origin from the total read population [10–14, 16, 17].

Here we present a new open source software package, RiboTagger, (https://github.com/xiechaos/ribotagger) to analyze rRNA data from shotgun sequencing reads. The software takes raw metagenome or metatranscriptome sequencing reads in FASTQ or FASTA files as input, and is able to process billions of Illumina HiSeq reads under an hour. RiboTagger produces a BIOM formatted files for downstream analysis in standard packages like QIIME [18] or MEGAN [19]. It is equally sensitive over all known bacterial and archaeal phyla and classes, and highly specific in not classifying non-rRNA sequences as rRNA.

## Implementation

**Fig. 1:**
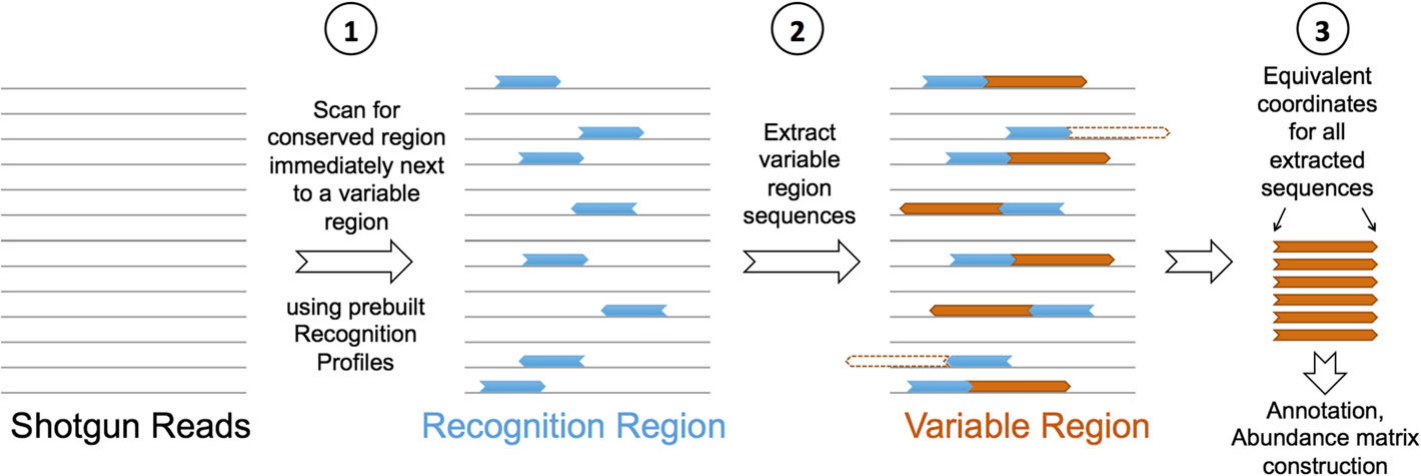
Schematic representation of RiboTagger detection scheme. Starting with shotgun sequencing reads from (either gDNA or cDNA) RiboTagger procedes as follows; (1) all reads are screened using PSSMs for the presence of a conserved recognition sequence (*blue arrowed rectangles*) adjacent to V-regions using a cohort of pre-defined recognition profiles; (2) for reads that are positive for recognition sequences, the adjacent V-region tag sequence is extracted, assuming if sufficient length is available (*dashed blue* rectangles denote in sufficient length in the tag sequence); and (3) for related tag sequences, equivalent coordinates are defined, prior to counting and annotation

**Fig. 2:**
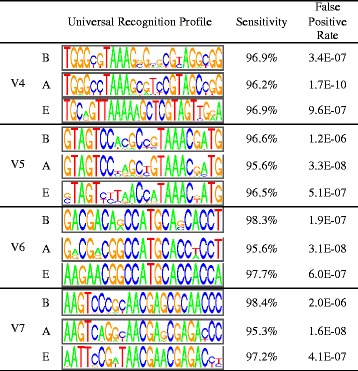
Universal recognition profiles for each of the variable regions V4--V7 used to target bacteria (B), archaea (A) and eukaryotes (E), respectively, with corresponding observed sensitivities and false positive rates

We start with genomic DNA or total RNA from a microbial community that has been sequenced, then attempt to recover sequencing reads covering a particular region on 16S rRNA gene using a short conserved *recognition sequence* (RS). A short sequence adjacent to the RS is used as the *tag sequence* (TS) to represent the origin of the 16S gene. For this strategy to be feasible with short-read sequencing technologies, by nature both RS and TS have to be short but informative and in particularly the RS must be conserved across all taxonomic branches, while the TS must be diverse with high taxonomic resolution. The boundaries of each hypervariable region are logical candidates for being RS, which we call *ribotags* from here on. After examining all bacterial and archaeal 16S sequences in the RDP database [20] we designed a combination of probe patterns and Position Specific Scoring Matrices (PSSM) to recognize the conserved site immediately outside a hypervariable region as RS, which we describe as a *universal recognition profile* (Fig. 1). The short region in the hypervariable region adjacent to the RS is then considered as a candidate tag sequence for the hypervariable region (Fig. 1). In this PSSM-based detection, the RS sequences are 23 n.t. in length and the TS are typically 33 n.t. in length. The lengths of both RS and TS were empirically determined so as to achieve a good trade-off between sensitivity and specificity, while maintaining a total length that is smaller than a typical sequencing read. For each of the three domains of life and each of the most commonly used variable regions, namely V4, V5, V6 and V7, we computed a universal recognition profile by analyzing the Greengenes database [21] (Fig. 2). To improve sensitivity, our implementation provides and can utilize a set of 17–30 supplementary taxon-specific recognition profiles for each of the variable regions.

## Results and discussion

### Validation analyses

Application of our universal profiles to the Greengenes database gave rise to a set of 90,061 ribotags. Each ribotag was assigned a taxon based on the set of SSU sequences that contain it, using the majority taxon in the case of discordance. The sensitivity of each of the profiles is over 95% percent (Fig. 2), as established by applying the profiles to all SSU sequences in the SILVA database [22]. To test the false positive rate, we ran the profiles on 5.6 billion faux reads, obtained by sampling all 80 nt non-overlapping fragments in the RefSeq database (release 66), calculating the false positive rate to be at most 2 × 10^−6^ in all cases (Fig. 2). To address the problem of the extent to which sequencing-error can generate false ribotags, RiboTagger estimates the expected frequency of each ribotag due to sequencing error, based on the observed frequencies of ribotags that differ by one letter and a simple probabilistic model.

**Fig. 3:**
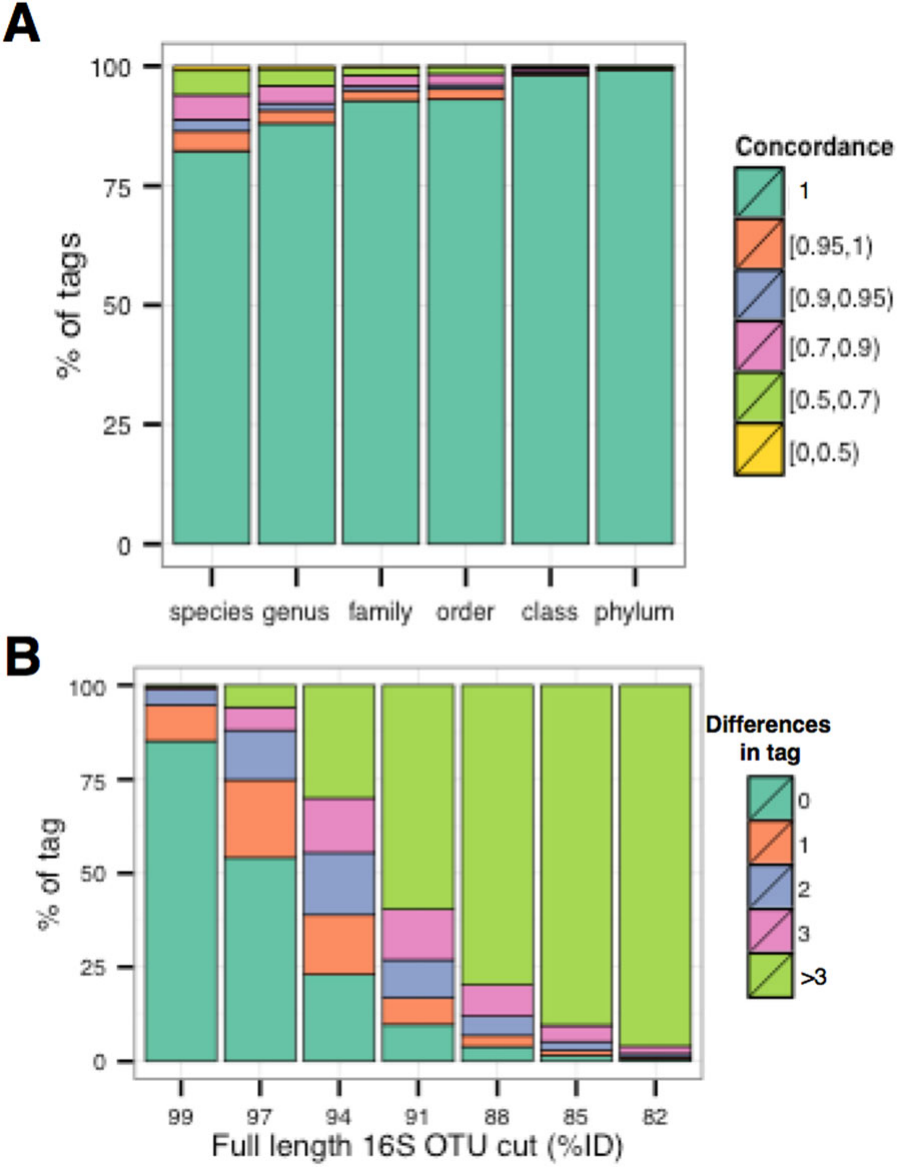
Percentage of V4 ribotags with a given range of concordance for different taxonomic ranks as (***a***) annotated in the Greengenes database and (**b**) as measured against Greengenes *de novo* OTU clusters as a function of cluster formation threshold

To determine how well ribotags can differentiate between organisms at a given taxonomic rank, we computed the *concordance* of all ribotags obtained from an analysis of the Greengenes database. We consider a ribotag as fully concordant (or to have concordance 1) at a given taxonomic rank, if all database sequences that contain that ribotag have the same taxonomic assignment at the given rank. For example, the concordance is between 0.95 and 1.00 if between 95 and 100% of the database sequences that contain the ribotag all have the same taxon assignment. The percentages of V4 ribotags with a given range of concordance (i.e. 1, [0.95,1), [0.95,0.90), etc.) for different taxonomic ranks as annotated in the Greengenes database are shown in Fig. 3a. Approximately 80% of all ribotags are fully concordant on the species level, while practically all reads are concordant at the phylum level. Results are similar for V6 ribotags, while the number of fully concordant ribotags is about 5% lower for V5 and V7 (data not shown). We also examined the degree of concordance against *de novo* OTU clusters defined by Greengenes over a range of percent identity thresholds, showing that for ribotags in clusters defined at 99% similarity, over 80% show no sequence differences (Fig. 3b).

Due to the short length of the 16S tags, we need to consider the possible influence of sequencing errors explicitly. PCR artifacts during library generation would lead to abnormally high numbers of reads with identical starting position and sequence content. Therefore, simply counting sequencing reads with different starting position can detect possible PCR artifacts. Using stringent quality value filtering for each nucleotide that covers 16S tags can also the impact of limit potential sequencing errors, which results extremely low error probability for each sequencing read considered. For example, with quality value threshold 30, the probability of sequencing error is only 0.1%. In addition, we also calculate the number of expected number of occurrence of any 16S tag, assuming the tag is not present at all. Assuming the tag of interest is not present, given the observed average sequencing error probability at each nucleotide position along the tag and the abundance of all other tag sequences with one nucleotide difference in the data, we can calculate the expected abundance of the tag of interest by sequencing error. Firstly, for the tag under study, all tag sequences with one nucleotide difference from the tag of interest are collected. Each of the 1 nt neighbors will contribute *E* = *n* × *e*/3 false positive counts to the tag of interest, where *n* is the number of reads covering the 1 nt neighbour and *e* is the observed error probability for the difference position on the tag of interest. The sum of *E* for all 1 nt neighbours gives the expected false positive tag of interest due to one sequencing error. Multiple sequencing errors can be also considered in a similar fashion but due to the quality-value filtering step, we can consider the probability of observing multiple sequencing errors in ribotag sequences as negligible.

### Example

To illustrate the results obtained from RiboTagger, we ran RiboTagger against a coupled DNA-Seq and RNA-Seq dataset generated from an activated sludge community of an operational wastewater treatment (Ulu Pandan Water Reclamation Plant, Northworks, Tank 3C), operated by the Public Utilities Board, Republic of Singapore; samples obtained between **10**/**08**/**2012** and **17**/**08**/**2012**). Genomic DNA and total RNA sequencing were each performed on a HiSeq2500 Rapid run using 250 bp paired end sequencing for DNA samples, and 150 bp paired end read sequencing for RNA samples. We observed a total of 4686 V4-region ribotags in the entire dataset. From the DNA dataset, we can estimate whether the number of observed 16S tags is as expected using the following rough approximation: if we assume there are up to 5000 genes in a typical free-living bacteria [23], and if we neglect the likely variation in 16S copy number across the member species in the community, and assume that one of those is a 16S gene with roughly 9 equally sized V-regions and 10 equally sized conserved regions, then we would expect approximately 308,505,950/(19 × 5000) = 3247 reads to originate from a single V-region in our analysis, which is a conservative underestimate to our observed number. For the RNA data, we report only results for one sample to for purposes of illustration, specifically with a total of 41,523,808 RNA reads available after QC filtering., we obtained a total of 4867 tags, of which the top 11 accounted for 20%, 80% and 95% of community membership (as described by total number of reads). Collectively there are 327 unique genera detected, 203 families, 128 orders and 51 phyla, The majority of annotated tags at kingdom level (1450) were attributable to bacteria, as expected in this community, with 34 and 40 tags being assigned to archaea and eukaryota, respectively. Approximately 3343 tags could not be assigned any annotation using SILVA v119 [22], including 1 and 41 in the top 20% and 50% of the community, respectively. These latter results highlight the substantial numbers of unknown taxa residing in complex microbial communities.

### Comparison to related programs

Using the RNA dataset described above, we compared the computational time of RiboTagger against two other existing search tools, RiboFrame [16] and SSUSearch [17], which are designed for retrieval of 16S sequences from whole metagenomic sequencing datasets. The comparison was carried out on a standalone server with the following specifications—2 Intel Xeon X7542 (18 M Cache, 2.66 GHz) CPUs, 128 GB memory, internal 146 GB HDD and external 8 TB RAID HDD. RiboTagger took approximately 90 min to complete, and we observed RiboTagger to be approximately 6 times faster than RiboFrame (~9 h) and 6.6 times faster than SSUSearch (~10 h). As RiboTagger and SSUsearch both used SILVA annotations, we compared the identity and relative abundance of detected phlya between both programs. In total 112 phyla were detected (51 with Ribotagger and 86 with SSUsearch) of which 25 were common between the two methods. The number of unclassifiable sequences differed, with 21.6% being called in SSUsearch and 47.5% in RiboTagger, however, of the 25 common phyla, 7 accounted for 95% of community composition in each set of results. The overall correlation between relative abundance was 0.99 (with 25 common phyla) and 0.88 (using all 112 phyla with non-detects set as zero). We note that while some of these differences may be accounted for by different database versions (SILVA v115 and SILVA v119 for SSUearch and Ribotagger, respectively) and/or handling of unclassifible sequences, these results suggest that further investigation of the differences between detection methodologies are warranted.

### Practical aspects

RiboTagger is a implemented as a platform independent Perl program. It can be executed in a single command in several modes with are briefly described here, along with their respective outputs. Full details can be found on the RiboTagger project page on Github (see Availability and Requirements)

### A. Single input file mode

The most basic way to run RiboTagger is using a single FASTQ file (including with either gzip and bzip2 compression), which will generate an output file formatted as a table with the following fields:

- **tag**: the tag sequence for the variable region
- **n**: the number of reads that contains this tag
- **npos**: the number of different locations of the tag on their source reads (a large value of **n** and a small value of **npos** indicates the presence of duplicated reads or would be observed if applied to amplicon sequencing reads).
- **fp**: the number of reads you would expect to see this tag due to sequencing errors alone
- **long.total.count**: the number of reads containing a longer sequence of this tag (see the-long option)
- **long1.count**, **long2.count**, **long3.count**: number of reads containing the most abundant variants of this tag’s long sequences (low long1.count/long.total.count ratio indicates that this tag is very likely representing a mixture of “species”)
- **long1**, **long2**: the most abundant long representive sequences of this tag

### B. Multiple input file mode

For multiple input files, RiboTagger can return a series of files. Data from paired end read data can also be combined into a single output. The.tab file returns a table of unnormalized read counts, with ribotags indexed in rows and samples indexed in columns. The.anno files contains QC, metadata and annotations, if available, for the same set of ribotags with the following columns fields:

- **tag**: the ribotag sequence
- **use**: “tag” or “long”, whether the annotation was based on the short tag or long representative sequence
- **taxon**_**level**: taxa rank of this annotation of this tag
- **taxon**_**data**: taxa rank of the most specific annotation appeared in the database (SILVA or Greengenes) for this tag
- **long**: the long representative sequence of this tag
- **long**_**total**: the number of samples having any long representative sequence
- **long**_**this**: the number of samples having this long sequence as its major representative of this tag
- **support**: the number of database sequences having this tag or long sequence
- **confidence**: the proportion of the database sequences agreed on this annotation
- **k**, **p**, **c**, **o**, **f**, **g**, **s**: annotation for each of the taxa ranks, namely kingdom/domain, phylum, class, order, family, genus, and species

The *.xls file is an Excel file combining data from the *.tab and *.anno files. Using biom.pl, a *.biom file can be subsequently used by QIIME [19]. We have implemented options for generating annotations to either SILVA [24] or Greengenes [21]. All 4 files types can automatically be generated if RiboTagger is run in batch mode.

## Conclusions

Here, we have developed software for the fast recovery of SSU-RNA sequences from shotgun nucleic acid surveys of complex microbial communities. Our code is fast, completing an analysis of about 40 M reads within 1.5 h, and will output an annotated matrix of read counts that can be used for downstream community profiling analysis with minimal further processing. Our implementation executes in a single line, avoiding the complications and the lack of robustness inherent in combination-type pipelines and is at least 6 times faster than SSUsearch [17] and RiboFrame [16]. Additional, we also note that our approach avoids the use of OTU generation, which recent analyses suggest may carry significant advantages in resolving intra-community dynamics for some classes of experimental design, such as time series experiments [25].

## Availability and requirements

**Project name**: RiboTagger

**Project home page**: https://github.com/xiechaos/ribotagger

**Operating system** (**s**): Platform independent

**Programming language**: Perl

**Other requirements**: None

License: GPLv3

**Any restrictions to use by non**-**academics**: none.

## Abbreviations

OTU: Operational taxonomic unit
PSSM: Position specific scoring matrix
RS: Recognition sequence
TS: Tag sequence
